# Comparison of food extraction techniques and impact of nitrogen fertilization on the potential allergenicity of soybean related to birch pollen-food allergy syndrome

**DOI:** 10.3389/falgy.2025.1650232

**Published:** 2025-09-23

**Authors:** Paulien Verscheure, Robin Daelemans, Lieve Coorevits, Laura Van Gerven, Raf Aerts, Rik Schrijvers

**Affiliations:** 1 Department of Microbiology, Immunology and Transplantation, Allergy and Clinical Immunology Research Group, KU Leuven, Leuven, Belgium; 2 Department of Biology, Division Ecology, Evolution and Biodiversity Conservation, KU Leuven, Leuven, Belgium; 3 Department of General Internal Medicine, Division of Allergy and Clinical Immunology, University Hospitals Leuven, Leuven, Belgium; 4 Department of Otorhinolaryngology, Head & Neck and Surgery, University Hospitals Leuven, Leuven, Belgium; 5 Department of Neurosciences, Experimental Otorhinolaryngology, Rhinology Research, KU Leuven, Leuven, Belgium; 6 Risk and Health Impact Assessment, Sciensano (Belgian Institute for Health), Brussels, Belgium

**Keywords:** allergy, birch pollen, plant-based foods, nitrogen, birch pollen-food allergy syndrome

## Abstract

**Background:**

Birch pollen-food allergy syndrome is triggered by cross-reactive allergens in plant-based foods. Environmental factors such as nitrogen fertilization may influence food allergenicity, but this has not been studied before.

**Methods:**

We compared and optimized protein extraction protocols for birch-homologue foods, including apple, carrot, and soybean. Various extraction buffers and mixing methods were tested for consistency and protein yield. We applied this to a pilot study assessing potential changes in the allergenic potential of plant-based foods due to altered nitrogen availability. A greenhouse experiment was conducted in which soybean plants were subjected to different nitrogen fertilization treatments. Allergenicity was evaluated using *ex vivo* basophil activation testing in five individuals with birch pollen-food allergy syndrome.

**Results:**

No major differences were observed between the tested extraction protocols, and key allergens were detectable in all food sources. In the pilot experiment, fertilized soybeans showed visible changes in size, a smaller shape, a different protein profile, and lower basophil reactivity compared to unfertilized soybeans.

**Conclusion:**

Our findings support the feasibility of standardized extraction methods. Varying nitrogen fertilization in soybeans resulted in altered physical, proteomic, and allergenic characteristics in this pilot study. Our results highlight the need for further research on environmental influences on food allergy.

## 1 Introduction

Pollen-food allergy syndrome (PFAS) is a common manifestation of food allergy in individuals sensitized to airborne pollen allergens. It was referred to as oral allergy syndrome (OAS) due to the typical presence of oropharyngeal symptoms, but was redefined as PFAS to reflect its potential for more severe systemic reactions in a subset of patients (1, 2). In contrast to classical primary food allergies, where primary sensitization typically occurs via the gastrointestinal (GI) tract and symptoms often occur systemically, PFAS arises from primary sensitization to airborne allergens (e.g., the major birch pollen allergen Bet v 1). This sensitization leads to IgE-mediated cross-reactivity with structurally homologous proteins present in various plant-based foods (2). This cross-reactivity is highly dependent on structural similarities between pollen and food allergens, particularly among highly conserved protein families like Bet v 1 homologues (e.g., Mal d 1 in apples, Dau c 1 in carrots, Gly m 4 in soybeans) (1, 2). These proteins are heat-labile and susceptible to digestion, as a result of which they typically only cause mild oral symptoms, while more stable proteins like nonspecific lipid transfer proteins (nsLTP) are more likely to trigger systemic reactions (3).

In northern and central Europe, birch pollen (BP) represents the predominant allergenic tree pollen, and approximately 70% of birch pollen-allergic individuals suffer from allergic symptoms upon ingestion of cross-reactive foods (1, 4, 5). Clinical manifestations generally occur immediately after food ingestion (within 5–15 min), and reactions are usually mild and localized to the oropharyngeal region, presenting as itching, tingling, or swelling (i.e., angioedema) of the mouth, lips, tongue, and throat (2–4). However, patients might also rarely present with severe systemic reactions (3). Approximately 3% of patients with PFAS experience systemic symptoms without any oral symptoms, and 1.7% even experience anaphylactic shock (2).

Recently, increasing levels of anaphylaxis to plant foods in the context of PFAS have been reported, with some studies citing incidences as high as 8.9% to 12% (6–8). The exact cause of these more severe presentations remains enigmatic and may include various factors. Generally, the most accepted risk factors include the circumvention of gastric digestion by labile food allergens, enabling structurally intact proteins to reach distal regions of the GI tract (9). In addition, several cofactors, such as exercise, alcohol intake, fasting, and the use of NSAIDs, are known to increase allergen uptake and exacerbate allergic symptoms (10). Fasting accelerates gastric emptying and allows rapid passage of foods (9). The use of antacid medication (e.g., proton pump inhibitors) limits the degradation of labile food allergens (9). Additionally, the intake of large quantities of allergenic foods over a short period may elevate the risk for severe reactions (9). Nevertheless, the intrinsic allergen content of food substances could play a role. Furthermore, individual variability in reactivity to PR-10 proteins may render certain patients more susceptible to severe systemic responses.

At the University Hospitals Leuven, a relatively large group of patients experiencing anaphylaxis due to BP-related food allergies was identified. This observation prompted an interest in potential factors contributing to an increased allergenic potential of certain plant-derived food allergens. Specifically, we hypothesize that increased nitrogen availability during plant growth may alter the expression levels and/or allergenicity of relevant food proteins, potentially accounting for the observed rise in reaction severity. Therefore, the present study aims to explore different extraction protocols for plant-based food products and apply this to a pilot study evaluating the impact of environmental nitrogen enrichment on food allergy severity.

## 2 Materials and methods

### 2.1 Extraction methods

The extraction protocols were based on available literature for apple (*Malus domestica*) (11–14), carrot (*Daucus carota*) (15–18), and soybean (*Glycine max*) (19–21). We tested different tissue processing methods and various extraction buffers (Table 1), explained below.

**Table 1:** Extraction buffers.

Extraction buffer name	Buffer composition
Extraction buffer #1	PBS
Extraction buffer #2	PBS with 2 mM EDTA, 2% (w/v) PVPP, 4 mM DTT, 0.2 mM PMSF
Extraction buffer #3	PBS with 1 mM EDTA, 2% (w/v) PVPP, 4 mM DTT, 1 mM PMSF, 10 mM DIECA

EDTA, ethylenediaminetetraacetic acid; PVPP, solid polyvinylpolypyrrolidone; DTT, Dithiothreitol; PMSF, phenyl methyl sulfonyl fluoride; DIECA, sodium diethylthiocarbamate.

For apples, small apple pieces (with peel, freshly bought from the store or stored at 4°C for 4 days, Jonagold variety) were brought into solution and mixed with extraction buffers #1, #2, and #3, and for some with the addition of protease inhibitors (Supplementary Figure S1A). The first method for carrots was to cut them into small pieces and grind them with a coffee grinder. The second method was to put small carrot pieces into a solution and blend them with a mixer. The third method was to snap freeze the carrot pieces with liquid nitrogen and crush them into powder with a pestle and mortar (Supplementary Figure S1B). Extraction buffers #1, #2, and #3 were used, with the addition of protease inhibitors. For soybeans, sample processing was done by mixing dry soybeans into powder and bringing them into solution (method 1, Supplementary Figure S1C) or by soaking dry soybeans in the extraction buffer and mixing (method 2, Supplementary Figure S1D), both with extraction buffer #1 and #2.

Protein concentration was determined via the Bradford (B6916, Sigma-Aldrich, Saint Louis, Missouri, United States) and bicinchoninic acid (BCA) assay (23227, Thermo Scientific, Waltham, Massachusetts, United States). Four replicate measures were performed, outliers were removed, and the average was used to determine the protein concentration (µg/ml). Protein concentration was used for normalization in further experiments. Aliquots were stored at −20 °C until further use.

### 2.2 Protein extract characterization (SDS-PAGE)

Protein extract separation was done under reducing conditions using the MES SDS running buffer (NuPage, Invitrogen, Thermo Fisher Scientific). Separation was performed on a 10% Bis-Tris gel (NuPAGE, Invitrogen) at 160 V for 1 h. For protein visualization, a silver staining (Pierce Silver Stain kit, Thermo Fisher Scientific) was performed according to the manufacturer's protocol. The molecular weight (MW) of the proteins was estimated by comparison with an established protein marker (SeeBlue Plus2 Prestained Standard, Invitrogen).

### 2.3 Immunoblot

Following SDS-PAGE, proteins were transferred by electrophoretic transfer at 30 V for 1 h20 onto a polyvinylidene difluoride (PVDF) membrane (Immobilon-P, Merck, Darmstadt, Germany). Transfer buffer (Table 2) was used to facilitate protein blotting. Remaining free binding spots were blocked with PBST-5% non-fat dry milk (NFDM) for 1 h at RT. After washing 4 times (5 min each) with PBST, the membrane was incubated with patient serum (1/8 dilution) overnight at 4 °C. Next, after washing again 4 times (5 min each) with PBST, the membrane was incubated with mouse anti-human IgE (GTX27382, GeneTex, Irvine, California, United States) (1/1,000 dilution) for 1 h at RT. Followed by again washing 4 times (5 min each) with PBST, and incubation with secondary horseradish peroxidase (HRP)-labeled goat anti-mouse IgG (1/20,000 dilution) for 2 h at RT. Finally, after the last wash (4 times, 5 min each, with PBST), protein bands were detected with chemiluminescence (Western Lightning Plus ECL-kit, Perkin Elmer, Waltham, Massachusetts, United States) and visualized with ImageQuant LAS 500 (GE Healthcare Life Sciences, Chicago, Illinois, United States) or the Odyssey XF Imager (LICORbio, Lincoln, Nebraska, United States).

**Table 2:** Immunoblot buffers.

Buffer name	Buffer composition
Transfer buffer	31.2 mM Tris, 239.77 mM Glycine, 20% (v/v) Methanol
PBST	0.05% Tween20 (Sigma-Aldrich, Saint Louis, Missouri, United States) in PBS
Dilution buffer	1% Bovine Serum Albumin (BSA, Sigma-Aldrich) in PBS

### 2.4 Basophil activation test (BAT)

The basophil activation test (BAT) was performed according to our in-house protocol as described previously (22). Peripheral blood was collected in Lithium Heparin tubes (BD Vacutainer). Fresh blood samples were stimulated with various concentrations of our protein extracts diluted in basophil stimulation buffer (BSB; 20 mM Hepes, 133 mM NaCl, 5 mM KCl, 7.5 mM CaCl2, 3.5 mM MgCl2, 1 mg/ml HAS, 0.5 mM Glucose, pH 7.4, supplemented with 60 ng/ml IL-3) for 25 min at 37 °C. For the boiled conditions, samples were put in boiling water for 30 min before basophil stimulation. Positive controls included anti-human IgE (50 ng/ml in BSB, Sigma-Aldrich, Saint Louis, Missouri, United States) and N-Formylmethionine-leucyl-phenylalanine (fMLP, 40 nM in BSB, Sigma-Aldrich). BSB was used as a negative control. Cells were stained with Phycoerythrin (PE) anti-human CD123 (Clone 6H6, 5 µg/ml), Alexa Fluor (AF) 647 anti-human HLA-DR (Clone L243, 5 µg/ml), Fluorescein isothiocyanate (FITC) anti-human CD63 (Clone H5C6, 20 µg/ml) (all BioLegend, San Diego, California, United States). Basophils were gated as CD123^+^/HLA-DR^−^ cells, and CD63 expression was used as a measure for basophil degranulation or activation. Samples were acquired using the LSR Fortessa flow cytometer (BD Biosciences, Franklin Lakes, New Jersey, United States) and analyzed with FlowJo v10.8.1 (FlowJo LLC, BD Biosciences).

### 2.5 Soybean greenhouse experiment

Soybean plants (*Glycine max*, commercial variety “Tasso”) were grown in a commercially available seedling substrate (DCM product code 1004525, <1 kg NPK/m^3^), characterized by a low nutrient content to minimize salt stress and promote root development in early growth stages. The plants were cultivated under controlled greenhouse conditions and were subjected to different fertilization treatments (NPSOY) starting 2 weeks after sowing. A total of four conditions were established, with eight plants per condition. The unfertilized control condition (1) only received tap water, the moderately fertilized condition (2) was treated with tomato fertilizer, the highly fertilized condition (3) was treated with tomato fertilizer with additional NH_4_NO_3_, and the very highly fertilized condition (4) was additionally treated with extra NPK (nitrogen, phosphorus, potassium) granules (Supplementary Table S1). These conditions corresponded to estimated nitrogen input levels of 0 kg N/ha (Condition 1), 25 kg N/ha (Condition 2), 50 kg N/ha (Condition 3), and 75 kg N/ha (Condition 4), respectively. For reference, typical nitrogen application rates in soybean cultivation range between 25 and 50 kg N/ha under field conditions. Beans were harvested, and a selection of 6 samples (3 from unfertilized and 3 from fertilized plants) was extracted and normalized for protein concentration (Bradford). This selection was based on sample volume and contained representative samples from the unfertilized an fertilized conditions. This selection (*n* = 6) was further used to determine the allergenicity in birch pollen-allergic adult patients suffering from PFAS (*n* = 5) via basophil activation testing.

### 2.6 Clinical study design and patient inclusion

Birch pollen-allergic adult patients suffering from PFAS were included in our analysis. Patients were recruited during routine consultations at the Allergy Department (UZ Leuven). Ethical approval for this study was provided by the Ethical Committee Research of UZ/KU Leuven (S65184), and informed consent was obtained from all patients. Allergies were confirmed via routine skin prick testing and allergen-specific IgE measurements (Supplementary Table S2). Additionally, a validated questionnaire was used for the diagnosis of PFAS (23). Whole blood samples were collected for basophil activation testing, and serum for further immunoreactivity testing. Two subjects were included for the optimization experiments (NP-opt), and five patients were included for the soybean greenhouse experiment (Supplementary Table S2). A negative control, not suffering from any allergies, was included to control for background reactivity of our extracts (Supplementary Figure S2).

### 2.7 Ethics approval statement

The study involving human participants was conducted in accordance with the Declaration of Helsinki. The study was reviewed and approved by the Ethical Committee Research of UZ/KU Leuven (protocol code S65184 and date of approval: 25/03/2022). The participants provided their written informed consent to participate in this study.

### 2.8 Statistical analysis

No statistical testing was performed for the optimization experiments. For the greenhouse pilot experiment, normality of the data was evaluated using the Shapiro–Wilk test. Differences between multiple groups were detected via one-way ANOVA, differences between two groups via (un)paired t-testing or its non-parametric alternative.

## 3 Results

### 3.1 Comparison of apple extraction methods

**Figure 1:**
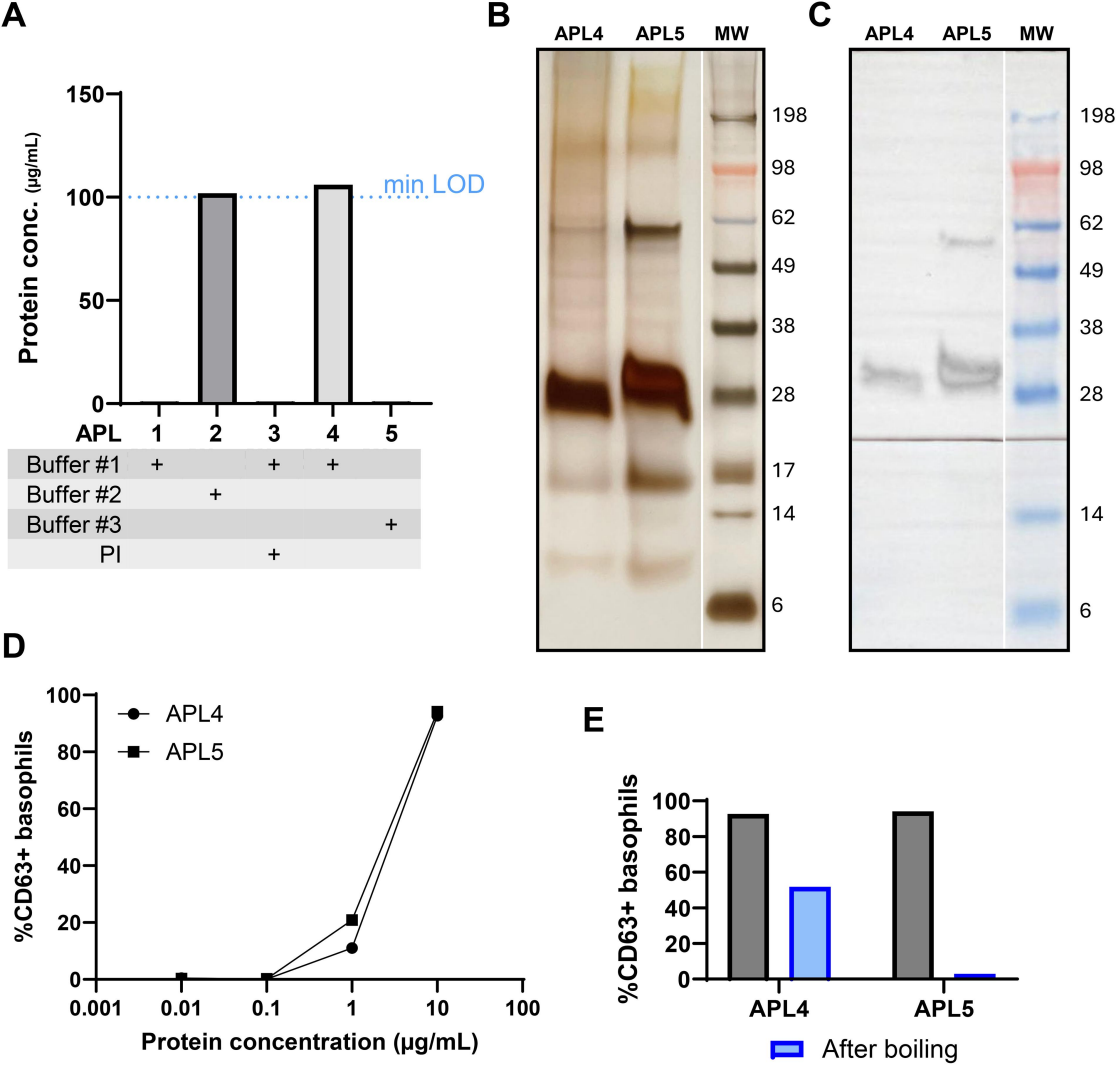
Comparison of apple extraction methods. **(A)** The protein concentration of apple extract was determined using the Bradford assay for the different extraction techniques employed. **(B)** Characterization of protein profile on SDS-PAGE. The same amount of extract was loaded for both samples. **(C)** sIgE reactivity of a BP-allergic individual suffering from apple cross-reactivity symptoms (subject: NP-opt1). The black line indicates the site where the membrane was physically sectioned to ensure proper serum incubation; the white lines indicate where the original images were digitally sectioned and reassembled. **(D)** Basophil activation test (BAT) on different apple extracts to determine differences in reactivity (subject: NP-opt1). **(E)** Comparison of BAT reactivity of apple extracts after boiling to diminish PR10 reactivity. APL, apple extract; PI, protease inhibitor; LOD, limit of detection; MW, molecular weight.

The protein extraction protocols for apples were compared for three different extraction buffers and the use of protease inhibitors. Only two out of the five extracts yielded a protein concentration just above the detection limit (Figure 1A, APL2 and APL4). APL4 (106 µg/ml) and APL5 (<100 µg/ml) were used for further optimization as these conditions were most consistently supported in literature. SDS-PAGE analysis (Figure 1B) revealed a limited protein profile including a protein band around 17 kDa, corresponding to the Bet v 1 homologue Mal d 1, a band around 28 kDa, although slightly different, possibly corresponding to the 23 kDa Thaumatin-like protein Mal d 2, and a band around 9 kDa corresponding to the nsLTP protein Mal d 3. Immunoblot analysis with serum of a BP-allergic individual suffering from oral allergy symptoms when eating raw apples was performed (Figure 1C). However, no IgE-reactivity was detected towards the expected Mal d 1 protein. Basophil activation testing (Figures 1D,E) revealed similar reactivity towards both apple extracts, and boiling diminished the reactivity more strongly for the APL5 extract, suggesting a relevant contribution of heat-labile proteins (e.g., Mal d 1).

### 3.2 Comparison of carrot extraction methods

**Figure 2:**
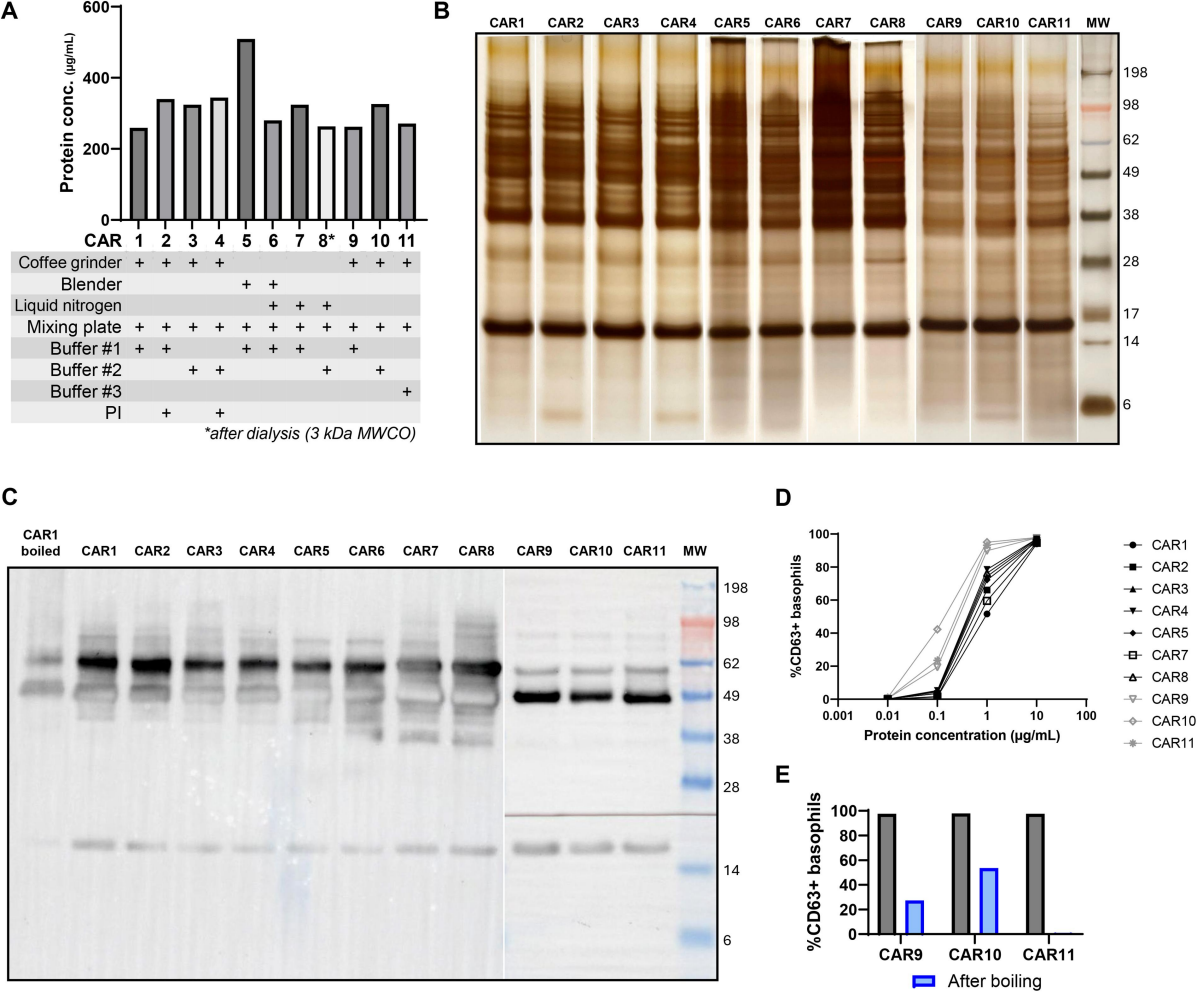
Comparison of carrot extraction methods. **(A)** Carrot extract protein concentration was determined using the Bradford assay for the different extraction techniques used. **(B)** Characterization of protein profile on SDS-PAGE. The white lines indicate where the original images were digitally sectioned and reassembled (lanes 1–8: CAR1 – CAR8 and lanes 9-11: CAR9 – CAR11 were performed on a separate gel with different developing times). **(C)** sIgE reactivity of BP-allergic individuals suffering from carrot cross-reactivity symptoms (lane 1–9: NP-opt2; lane 10–12: NP-opt1). The black line indicates the site where the membrane was physically cut to ensure proper serum incubation. **(D)** Basophil activation test (BAT) on different carrot extracts to determine differences in reactivity (subject NP-opt1; black: experiment 1; grey: experiment 2). **(E)** Comparison of BAT reactivity of carrot extracts after boiling to diminish PR10 reactivity. CAR, carrot extract; PI, protease inhibitor; MW, molecular weight.

The protein extraction protocol for carrots was compared for three different extraction buffers and the use of protease inhibitors (Figure 2A). All extracts yielded a similar protein concentration between 200 and 400 µg/ml. The CAR5 extract, made by just blending carrots in PBS, yielded the highest protein concentration of 509 µg/ml. SDS-PAGE analysis (Figure 2B) revealed a diverse protein profile, including a protein band around 17 kDa, corresponding to the Bet v 1 homologue Dau c 1, a band around 28 kDa, although slightly different, possibly corresponding to the 33 kDa Isoflavone reductase-like protein Dau c 5. In some extracts (i.e., CAR1, CAR5, CAR7, CAR9, and CAR10), a band around 14 kDa is visible, which corresponds to the profilin protein Dau c 4. Immunoblot analysis (Figure 2C) with serum of a BP-allergic individual suffering from oral allergy symptoms when eating raw carrots revealed IgE-reactivity towards the expected Dau c 1 protein, which was diminished after prolonged boiling of the extract. However, reactivity was rather limited, and also targeted other proteins with no known allergenic relevance. Basophil activation testing (Figures 2D,E) revealed limited variability between the different carrot extracts. Boiling diminished the reactivity of CAR9 and CAR10 and completely reduced CAR11 reactivity, again suggesting a relevant contribution of heat-labile proteins (e.g., Dau c 1).

### 3.3 Comparison of soybean extraction methods

**Figure 3:**
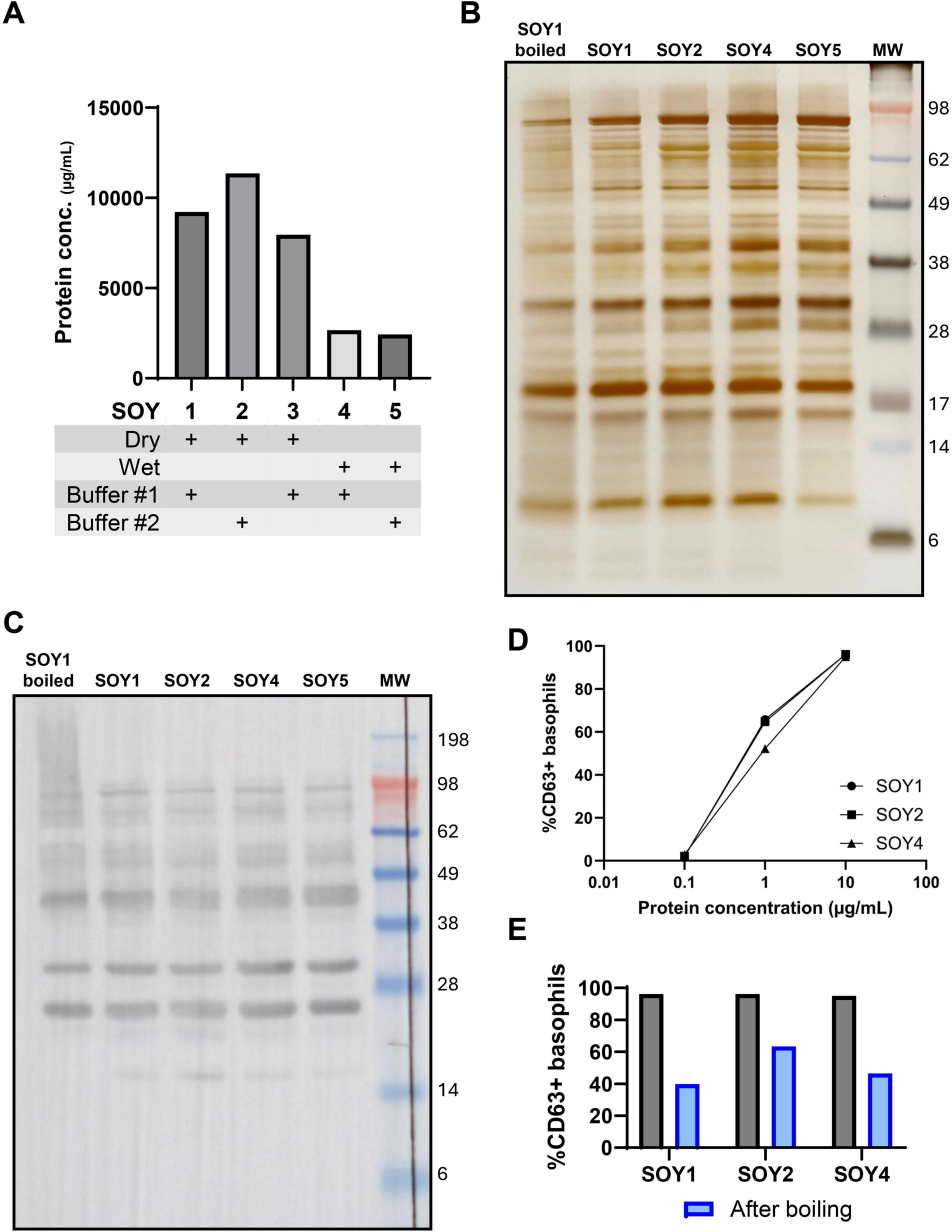
Comparison of soybean extraction methods. **(A)** Soybean extract protein concentration was determined using the Bradford assay for the different extraction techniques used. **(B)** Characterization of protein profile on SDS-PAGE. **(C)** sIgE reactivity of BP-allergic individuals suffering from soybean cross-reactivity symptoms (subject: NP-opt1). The black line indicates the site where the membrane was physically sectioned to ensure proper serum incubation. **(D)** Basophil activation test (BAT) on different soybean extracts to determine differences in reactivity (subject NP-opt1) and **(E)** comparison of BAT reactivity of soybean extracts after boiling to diminish PR10 reactivity. SOY, soybean extract; MW, molecular weight.

The protein extraction protocol for soybeans was compared for two processing methods and two extraction buffers (Figure 3A). Dry processing of the soybeans in a coffee grinder till soy flour was practically the most optimal method and yielded a higher protein concentration compared to the wet processing method. SDS-PAGE analysis (Figure 3B) revealed an extensive protein profile including a protein band around 17 kDa, corresponding to the Bet v 1 homologue Gly m 4. Additional proteins include Gly m 1 or Gly m 2, around 7 or 8 kDa, the profilin protein Gly m 3, around 14 kDa, the Gly m 5 and Gly m 6 subunits between 20 and 65 kDa, Gly m 7, around 76.2 kDa, and Gly m 8, around 28 kDa. Immunoblot analysis (Figure 3C) with serum of a BP-allergic individual suffering from oral allergy symptoms when eating soybeans was performed. However, only limited IgE-reactivity was detected towards the expected Gly m 4 protein, but this reactivity was diminished after prolonged boiling of the extract. Basophil activation testing (Figures 3D,E) revealed similar reactivity towards the three tested soybean extracts, and boiling diminished the reactivity of all three extracts, again suggesting a high contribution of heat-labile proteins (e.g., Gly m 4).

### 3.4 Impact of nitrogen enrichment on soybean allergenicity in the context of birch pollen-associated food allergy syndrome

**Figure 4:**
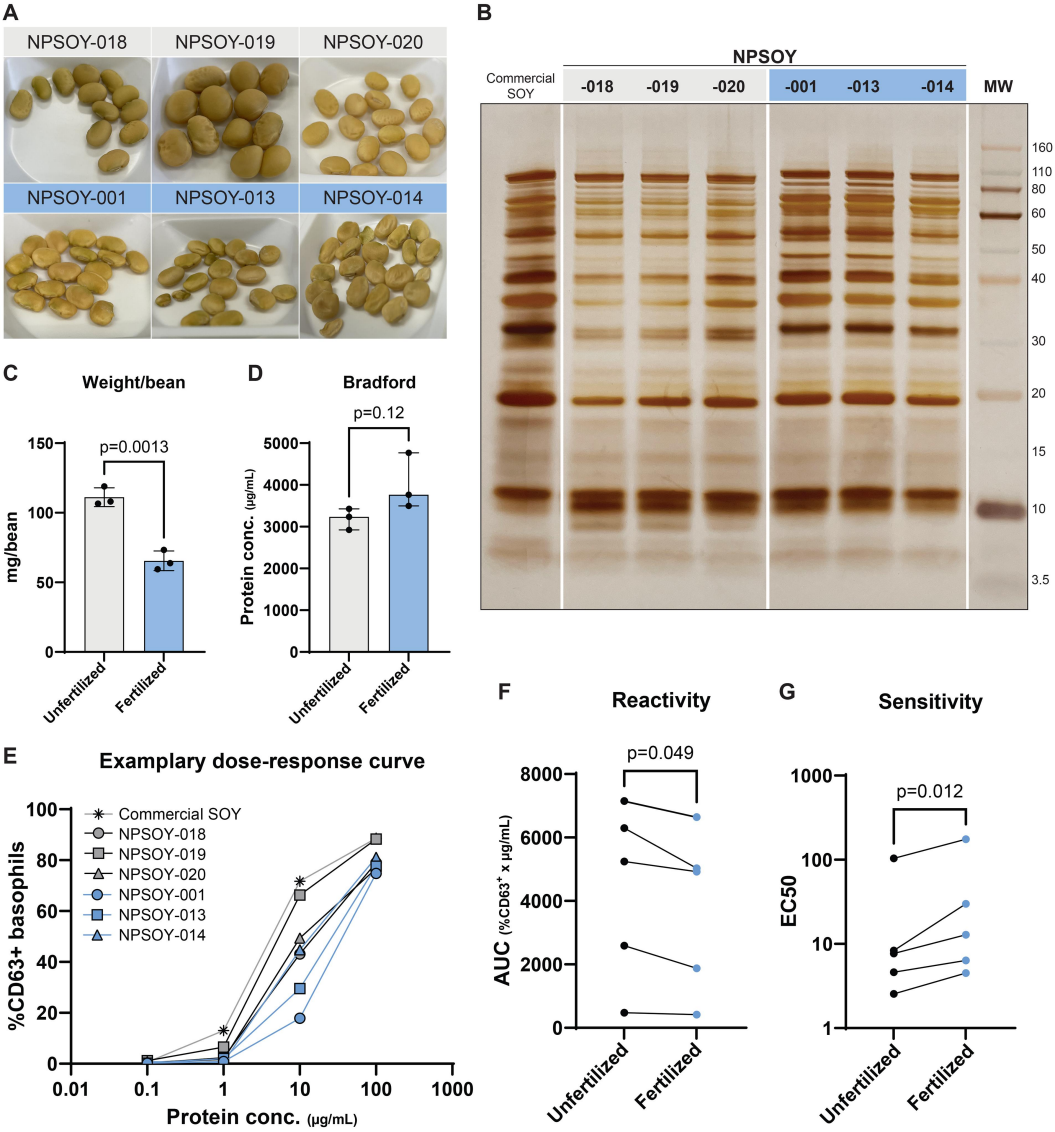
Soybean greenhouse fertilization experiments **(A)** pictures of soybeans used for total soluble protein extracts. Unfertilized samples are shown in grey, and fertilized samples are shown in blue. **(B)** SDS-PAGE and silver stain of the soybean extracts. The white lines indicate where the original images were digitally sectioned and reassembled. **(C)** Weight (mg) per bean between the unfertilized (U) and fertilized (F) group (*p* = 0.0013, unpaired t-test). **(D)** Total soluble protein concentration (Bradford) between the U and F group (*p* = 0.1211, Unpaired t-test). **(E)** Exemplary dose-response curve of basophil activation of 1 patient included in the study (NP164), measured as %CD63 + basophils (CD123 ^+^ HLA-DR^−^; *y*-axis) for different protein concentrations of the soybean extracts (µg/ml; *x*-axis). **(F)** Basophil reactivity (AUC *p* = 0.049, paired t-test) and **(G)** sensitivity (EC50 *p* = 0.012, paired t-test of log10-transformed data) between the U and F groups.

Soybean plants were cultivated under different fertilization treatments. Soybeans were collected, and we saw no differences in the amount of harvested soybeans per condition (Supplementary Figure S3). A selection of six beans (including 3 from the unfertilized control condition and 3 from the fertilized conditions) was used for further testing. We observed a visual difference in the beans between the two fertilization conditions. Beans from the unfertilized (U) condition were bigger, while beans from the fertilized (F) condition appeared smaller and shriveled (Figure 4A). The weight per bean was significantly lower for the beans from the fertilized conditions (Figure 4C, *p* = 0.0013). Soybean extracts were made, and total soluble protein concentration was determined. Protein concentration (Bradford) was not significantly different between the two conditions, but trended towards higher values in the fertilized beans, despite their smaller size (Figure 4D, *p* = 0.1211). On SDS-PAGE, we observed a difference in the protein profile of our soybean extracts (Figure 4B), where the unfertilized condition showed some additional protein bands, around 30 kDa and 8 kDa, compared to the fertilized condition. Five patients were included for basophil activation testing. We measured lower basophil reactivity or sensitivity towards the beans from the fertilized conditions (Figures 4F: AUC *p* = 0.049, and 4G: EC50 *p* = 0.012).

## 4 Discussion

For the first part of this study, we aimed to standardize the protein extraction protocol suitable for various food sources, with the goal of further use in allergy research. Numerous extraction protocols have been reported in literature, prompting us to evaluate a combination of different approaches (11–13, 15–21). We assessed the resulting extracts in terms of protein yield, allergen composition, and IgE reactivity in BP-allergic individuals with PFAS. Overall, except for some minor differences, the differences in total protein yield, content, and allergic reactivity were limited, suggesting that several extraction methods may be comparably effective for downstream allergenic analysis.

Among the tested food sources, apple presented the greatest challenge in achieving sufficient protein recovery. This is consistent with the naturally low protein content of apples. Generally, 1 g of fresh apple contains 1–30 µg of Mal d 1 protein, which may increase up to 100 µg after storage (24). In our extraction protocol, apples were used freshly, when bought from the store, or after 4 days of storage at 4°C. A possibility to increase the protein and Mal d 1 content would be to increase storage times under the correct conditions. Previous research has shown that this can significantly enhance protein content, with even a 13 times increase in Mal d 1 content after 12 weeks of storage for the Jonagold variety (25). Another limitation of our apple extractions would be that we performed them exclusively with Jonagold apples. Previous studies have shown that the type of apple cultivar can influence the Mal d 1 content of extracts (13, 26). The Jonagold cultivar is shown to have a generally low percentage of Mal d 1, which might also explain our observations. To ensure that the extraction protocol yields a reliable and sufficient amount of Mal d 1, future optimization should include comparison across different cultivars. Lastly, we observed clear basophil activation in response to both of our apple extracts, despite the absence of detectable IgE-binding activity on immunoblot. The underlying reason for this discrepancy remains unclear, but it might be due to differences between the two experimental approaches. While immunoblotting visualizes IgE binding to specific proteins, basophil activation testing evaluates the functional cellular response to allergen stimulation, which may capture additional mechanisms not revealed by protein-IgE reactions alone or masked by reducing SDS-PAGE conditions. Additionally, variability may also arise from differences in assay sensitivity, as basophil activation testing is often able to detect responses even in the absence of detectable IgE (27–29). Furthermore, we see a difference in heating resistance between the two tested apple extracts. Heating completely diminished basophil reactivity of APL5, while there is still visible reactivity towards the APL4 extract. According to literature, the heat sensitivity of Bet v 1-homologous allergens can be isoform-specific and pH-dependent (30). Therefore, observed differences can be explained by differences in extraction pH or isoform composition of the extracts.

For carrots, the protein concentration obtained was relatively consistent across different extraction techniques, with only subtle differences observed in the protein profiles of the different carrot extracts. Notably, the presumable Bet v 1 homologue Dau c 1 was detected at comparable levels in all extracts. Furthermore, allergic reactivity, as measured in a BP-allergic PFAS individual, was similar across these extracts, indicating that the various protocols did not substantially affect the immunoreactivity of Dau c 1. The differences in heat resistance between the three different carrot extracts can be explained similarly to those of the apple extracts described above (30). Notably, extracts prepared with extraction buffer #3 showed a complete inhibition of basophil reactivity after heating (for both apple and carrot), while the other extracts retained some residual reactivity.

Soybean extractions yielded consistently high protein concentrations, reflecting the naturally high protein content of this food. However, due to the complex allergen composition of soybeans, the proportion of the Bet v 1 homologue Gly m 4 was only estimated as 1% of the total allergenic protein fraction (31). However, basophil activation testing highlighted the presence of heat-labile proteins contributing to the allergenic response. The soybean proteins that are highly affected by heating include Gly m 4 and the profilin protein Gly m 3 (31). However, our subject NP-opt1 tested negatively towards the profilin allergen Bet v 2 (<0.35 kU_A_/L), meaning that the reactivity should mainly result from the Gly m 4 allergen.

In the second part, we conducted a soybean greenhouse experiment to determine the impact of nitrogen fertilization on soybeans, seed protein content, and allergenic potential. More specifically, we hypothesized that higher nitrogen availability during plant growth may alter the expression levels and/or allergenicity of relevant food proteins, potentially leading to differences in reaction severity. Supporting this hypothesis, Peñuelas et al. demonstrated an increase in immunogenic proteins in wheat by nitrogen intensification, and its possible impact on the rising prevalence of coeliac disease (32). Similarly, Stawoska et al. reported that increased nitrogen fertilization was linked to higher gluten content in wheat, potentially increasing the allergenic risk in gluten-sensitive individuals (33). Shi et al. described changes in the metabolite profiles of *Lycium barbarum* fruit, known as the goji berry, following nitrogen fertilizer application (34). Furthermore, although not representative of natural environmental exposure, *in vitro* nitration of food allergens has been shown to enhance their immunogenicity. Gruijthuijsen et al., for instance, observed a heightened allergic response towards *in vitro* nitrated ovalbumin, a major egg allergen (35). However, to date, no studies have directly tackled the impact of nitrogen fertilization on the allergenicity of plant-based foods implicated in PFAS.

In our pilot experiment, we observed no differences in the amount of beans harvested and the soybean protein concentration, but differences in protein profile could be observed. Contrary to literature and our hypothesis, basophil activation testing on five BP-allergic individuals with PFAS revealed significantly lower allergenicity towards soybeans from the fertilized conditions (32–34).

Previous studies have demonstrated that nitrogen fertilization can influence soybean seed yield and protein content, typically showing increased seed production and elevated protein concentrations in response to higher nitrogen availability (36, 37). Contrary to these findings, our results did not reflect such trends, suggesting that the nitrogen treatments applied may not have been completely effective in our specific experimental conditions. However, fertilized soybeans showed visible changes in size and shape compared to unfertilized controls. Additionally, the highest fertilization conditions might have been too high and might have impaired soybean plant growth, which might also be reflected in the size and shape of these fertilized soybeans (38). However, we observed alterations in specific protein profiles between unfertilized and fertilized soybean samples. This suggests that, with optimized greenhouse growth conditions, further investigation into the impact of nitrogen fertilization on soybean protein composition could offer valuable new insights into this area of research.

In conclusion, this study optimized and compared several protein extraction methods, showing no major differences in protein yield or allergen presence, supporting their suitability for food allergy research. While further optimization might be needed to increase the protein yield of protein-poor food products, our results provide a methodological base for this. Additionally, in our pilot greenhouse experiment, nitrogen fertilization produced minor variations in soybean size, shape, protein content, and allergenicity. These preliminary findings highlight the need for further studies with refined growing conditions to confirm and extend these results.

## Data Availability

De-identified data will be made available upon reasonable request from 30 days after the finish date of the NITROPOL project. Requests to access the datasets should be directed to the corresponding author, rik.schrijvers@uzleuven.be.
