# Supplementary material for: Comparison of food extraction techniques and impact of nitrogen fertilization on the potential allergenicity of soybean related to birch pollen-food allergy syndrome
Source: Front Allergy. 2025 Sep 23;6:1650232. doi: 10.3389/falgy.2025.1650232 (PMC12502077; doi:10.3389/falgy.2025.1650232)
Supplement: Supplementary file 1 [file Supplementaryfile1.docx]

Supplementary Material

# Supplementary Figures

**Figure S1. Pictures of the extraction method.** (A) Apple, (B) carrot, (C) soybean method 1, and (D) soybean method 2.

**A**


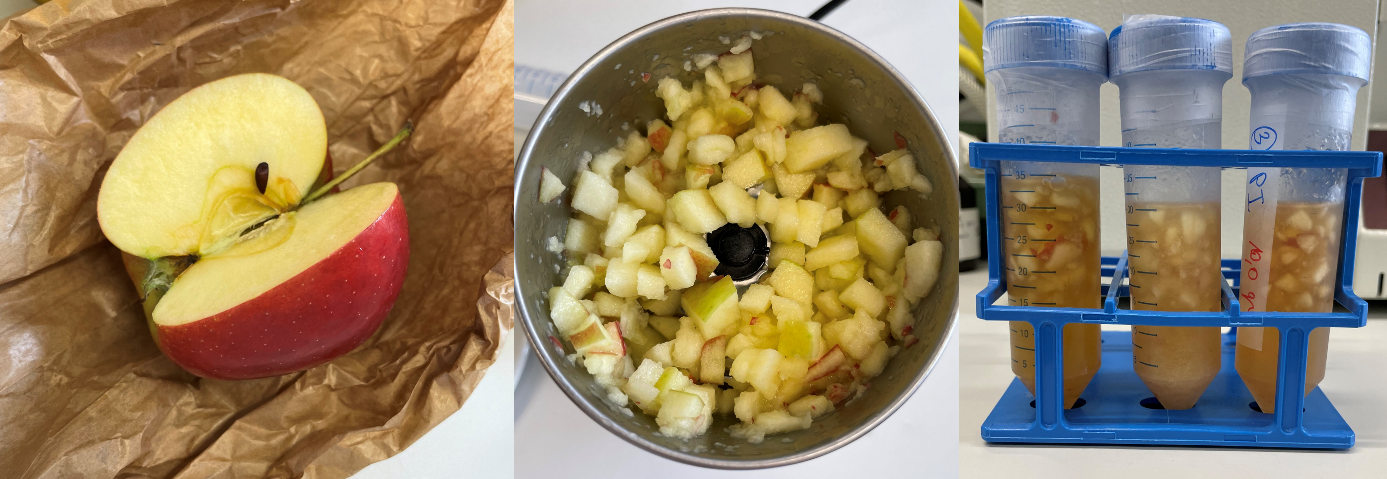


**B**


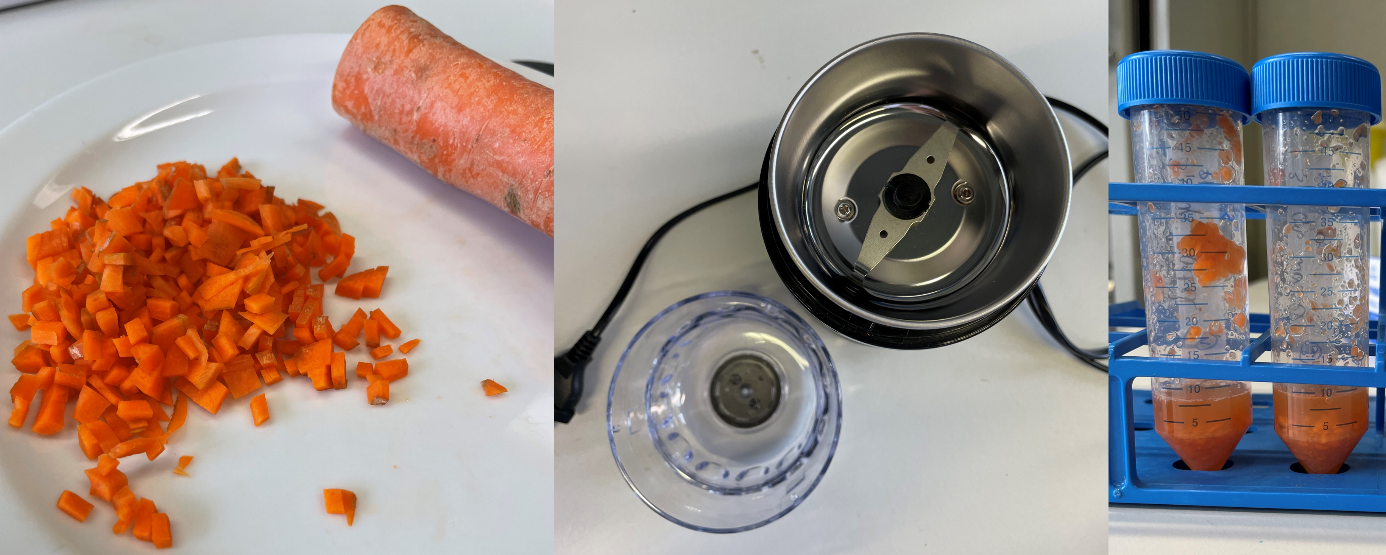


**C**


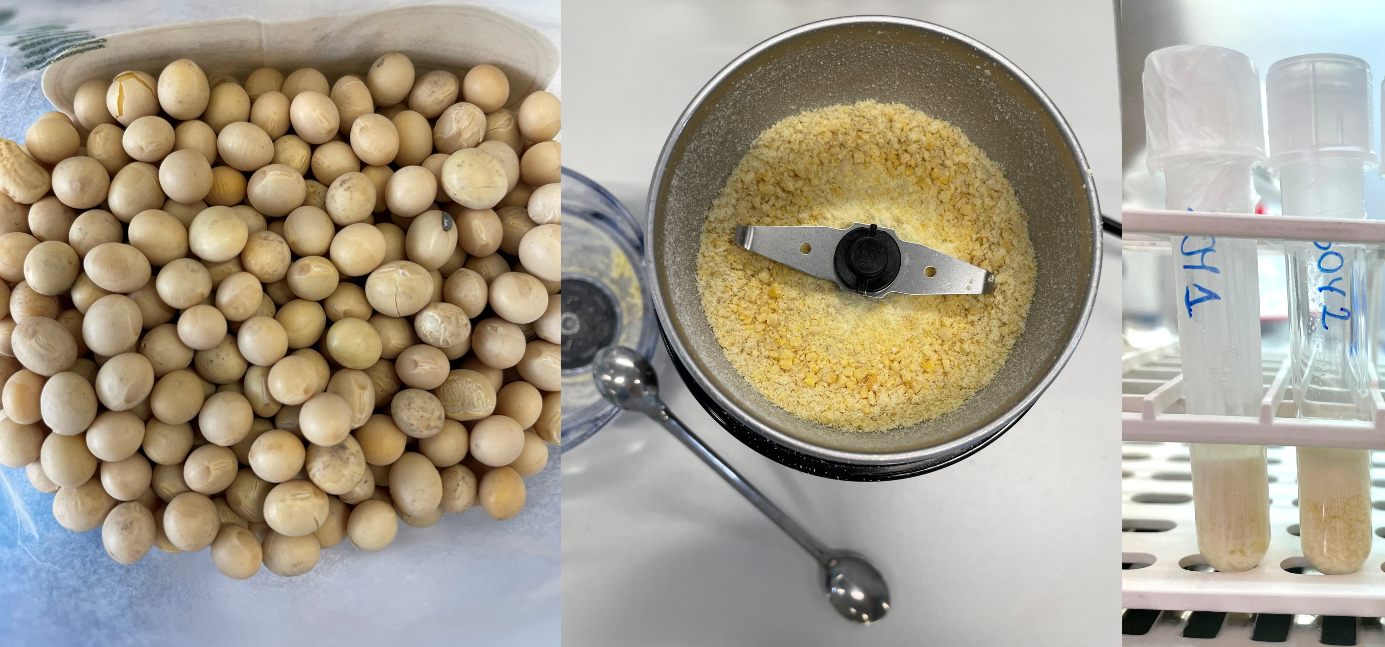


**D**


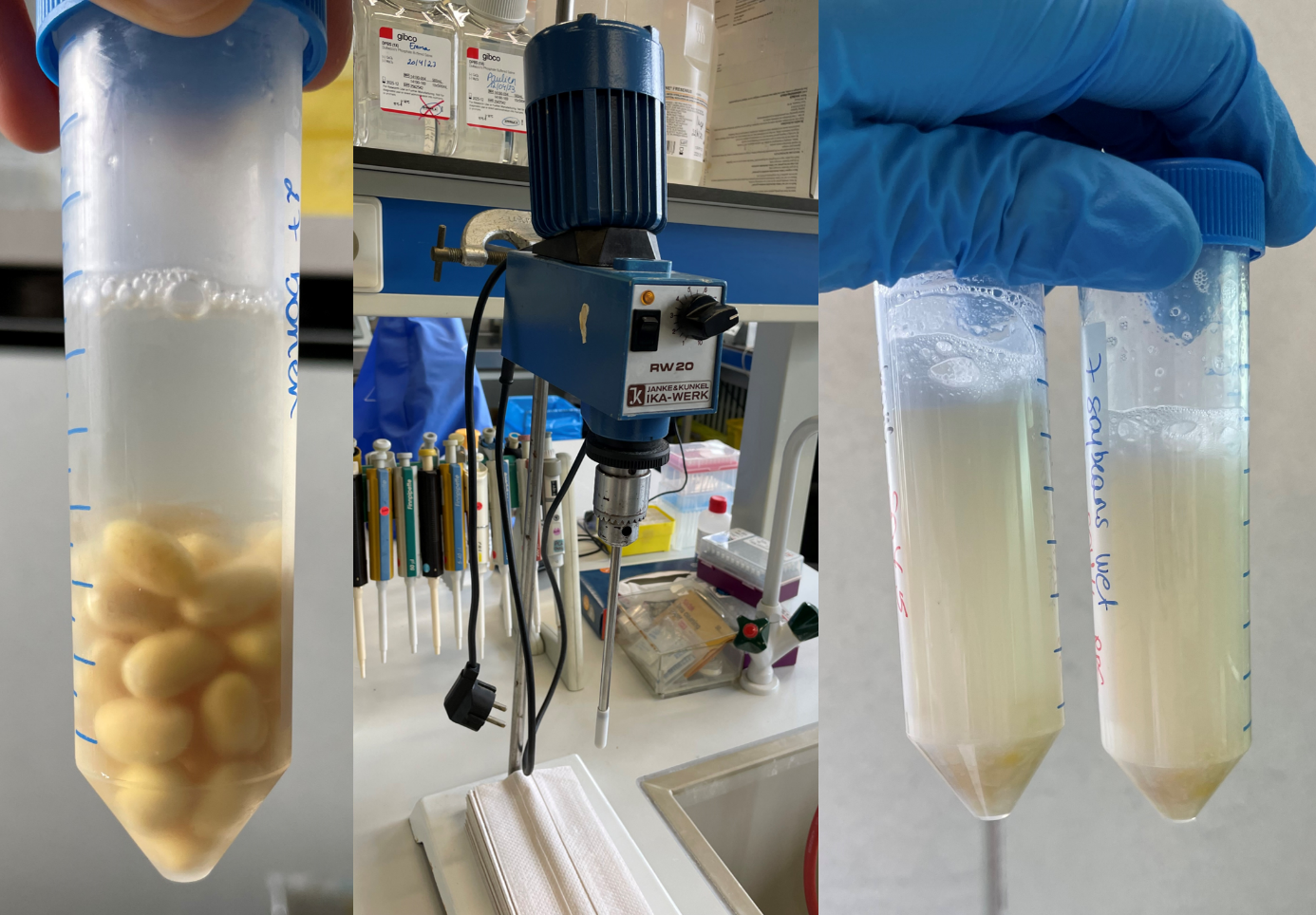


**Figure S2. BAT negative control.** Results of basophil activation test on a negative control subject not suffering from birch pollen allergies or any food allergies, including (A) controls, our (B) apple, (C) carrot, and (D) soybean extracts. Black represents experiment 1; grey represents experiment 2.

**
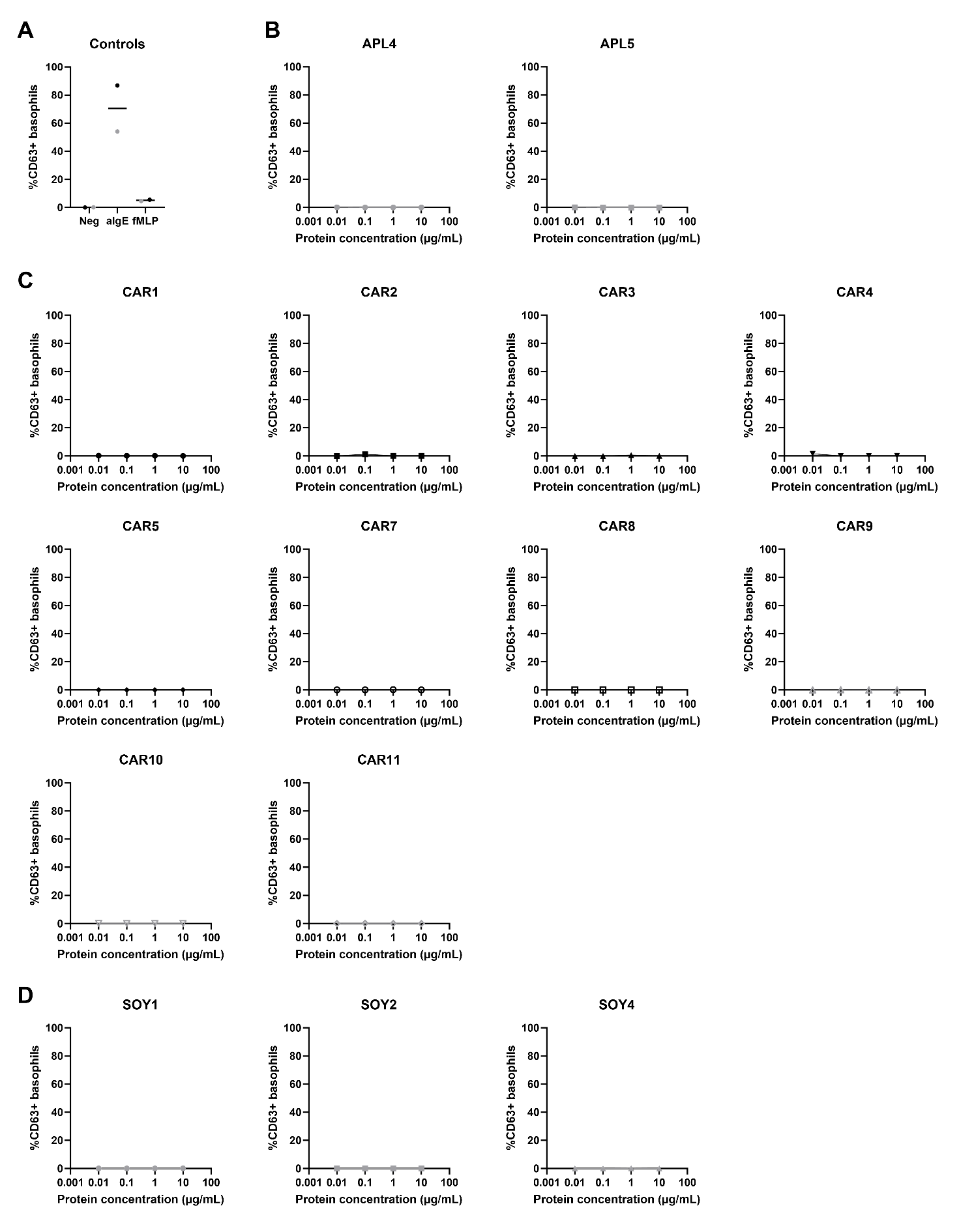
**

**Figure S3. Harvested soybeans from the greenhouse fertilization experiment.** Fertilization condition 1: no extra N (yellow), condition 2: moderate N (green), condition 3: high N (orange), condition 4: very high N (red). No significant differences between groups were observed. One-way ANOVA was used to determine significance.


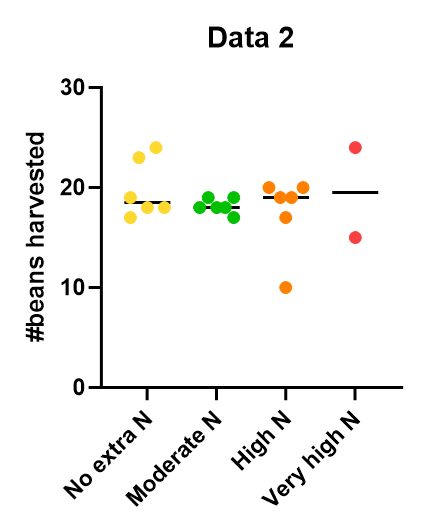


# Supplementary Tables

**Table S1. Fertilization conditions**

| **Fertilization conditions** | |
| --- | --- |
| No extra N (1) | Tap water |
| Moderate N (2) | Plants were treated with a two-part tomato fertilizer solution consisting of Solution A and Solution B, prepared with the following components per 100 liters of water:  Solution A:   - Calcium nitrate tetrahydrate (Ca(NO₃)₂·4H₂O): 15,349.75 g - Potassium nitrate (KNO₃): 3,286.08 g - Iron chelate (EDTA, 4.5%): 250 ml   Solution B:   - Potassium sulfate (K₂SO₄): 3,398.27 g - Magnesium sulfate heptahydrate (MgSO₄·7H₂O): 4,806.36 g - Monopotassium phosphate (KH₂PO₄, MKP): 2,653.76 g   These solutions were applied according to standard greenhouse practice, starting two weeks after sowing, and resulted in a cumulative nitrogen input equivalent to 25 kg N/ha. |
| High N (3) | In Condition 3, the same tomato fertilizer solutions were used as in Condition 2, supplemented with additional ammonium nitrate (NH₄NO₃) to reach a total nitrogen input of 50 kg N/ha. |
| Very high N (4) | In Condition 4, the same fertilization regime as in Condition 3 was applied, with the addition of granular NPK fertilizer (27:0:0) to further increase nitrogen availability, resulting in a total nitrogen input of 75 kg N/ha. |

**Table S2. Patient characteristics.** Overview of patient characteristics of subjects included for optimization and in the soybean greenhouse experiments. Results of routine skin prick testing with birch pollen (BP) and/or soymilk (Soy) and routine sIgE measurements with commercially available ImmunoCAPs (Phadia, Thermo Fisher) for birch pollen (t3), Bet v 1 (t215), soybean (f14), and rGly m 4 (f353). All patients were classified according to the validated questionnaire for the diagnosis of pollen-food allergy syndrome (PFAS). (1)

|  | SPT | | sIgE (kU_A_/L) | | | | Questionnaire-based PFAS classification |
| --- | --- | --- | --- | --- | --- | --- | --- |
|  | **BP** | **Soy** | **BP** | **Bet v 1** | **Soy** | **Gly m 4** |  |
| NP-opt1 | 3+ | 3+ | >100.0 | >100.0 | - | - | PFAS+ |
| NP-opt2 | 3+ | - | 8.25 | - | - | - | PFAS+ |
| NP134 | 3+ | - | - | 38.80 | 1.12 |  | PFAS + |
| NP137 | 3+ | 2+ | 6.53 | 5.39 | - | 1.25 | PFAS + |
| NP139 | - | - | 17.40 | 15.70 | - | - | PFAS + |
| NP164 | 3+ | - | 14.60 | - | - | - | PFAS + |
| NP185 | 3+ | 3+ | 10.60 | - | - | 2.51 | PFAS + |

# References

1. Skypala IJ, Calderon MA, Leeds AR, Emery P, Till SJ, Durham SR. Development and validation of a structured questionnaire for the diagnosis of oral allergy syndrome in subjects with seasonal allergic rhinitis during the UK birch pollen season. *Clinical & Experimental Allergy*. 2011;41(7): 1001–1011. https://doi.org/10.1111/j.1365-2222.2011.03759.x.
